# Exergaming to increase the exercise capacity and daily physical activity in heart failure patients: a pilot study

**DOI:** 10.1186/1471-2318-14-119

**Published:** 2014-11-18

**Authors:** Leonie Klompstra, Tiny Jaarsma, Anna Strömberg

**Affiliations:** Department of Social and Welfare studies, Faculty of Health Science, Linköping University, Linköping, Sweden; Department of Medical and Health Science, Division of Nursing, Faculty of Health Science, Linköping University, Linköping, Sweden; Department of Cardiology, County Council of Östergötland, Linköping, Sweden

**Keywords:** Active video game, Adherence, Exercise capacity, Exergame, Heart failure, Physical activity

## Abstract

**Background:**

Regular daily physical activity is recognised as important in heart failure (HF) patients, but adherence to physical activity is low (<50%). To improve adherence to exercise in HF patients, alternative approaches to motivate and increase self-efficacy to exercise are needed. Therefore, we have studied a new phenomenon: exergames (games to improve physical exercise). The aims of the study were to assess the influence of the exergame platform Nintendo Wii on exercise capacity and daily physical activity in heart failure patients, to study factors related to exercise capacity and daily physical activity, and to assess patients’ adherence to exergaming.

**Methods:**

A 12-week pilot study with a pretest-posttest design was conducted. The intervention consisted of an instruction on how to use the Wii and 12 weeks’ access to Wii at home. The main variables tested were exercise capacity (measured with a six-minute walking test), daily physical activity (measured with an activity monitor), and time exergaming (daily self-report with a diary). Bivariate correlations were used to assess associations between symptom experience, self-efficacy, motivation, anxiety, and depression.

**Results:**

In total, 32 heart failure patients were included. More than half of the patients (53%) significantly increased their exercise capacity after 12 weeks. No significant difference was found in daily physical activity between baseline and 12 weeks. Lower NYHA class and shorter time since diagnosis were factors significantly related to the increase in exercise capacity. The daily mean time spent exergaming was 28 minutes, and having grandchildren and being male were related to more time spent exergaming.

**Conclusion:**

Exergaming has the potential to increase exercise capacity in elderly, chronically ill cardiac patients. Although the daily physical activity did not change over time, exergaming was feasible for heart failure patients and might be a rehabilitation option for patients with heart failure.

## Background

Society faces an increasing number of chronically ill patients, of whom a considerable number are cardiac patients [1, 2]. Heart failure (HF) is a chronic condition that is often the end stage of other cardiac diseases and is known to have a poor prognosis. Most HF patients experience symptoms such as shortness of breath and fatigue, and report decreased physical capacity and quality of life [1]. HF patients also pose a considerable economic burden to the health care system due to frequent hospitalisations. Considering this impact of HF on patients and society, it is important to search for opportunities to improve outcomes. Regular daily physical activity is recognised to be important in HF patients [1, 3]. Guidelines on the treatment of HF recommend regular and structured physical activity, as this improves exercise capacity and quality of life, and may reduce mortality and hospitalisation in patients with mild to moderate chronic HF [1, 4]. Several studies have shown that physical activity, both home-based and hospital-based, is safe and beneficial for HF patients. The findings from a meta-analysis (ExTraMatch collaborative) suggested that patients randomised to physical fitness were less likely to be admitted to hospital and had a better prognosis [5]. These results were confirmed by the HF-ACTION (Heart Failure: A Controlled Trial Investigating Outcomes of Exercise Training) study, which showed a modest improvement in exercise capacity and decreased all causes of death in patients who exercised [6]. The main limitation of the HF-ACTION study was the poor adherence to the prescribed training regimen, with only 30% of the patients adhering to their exercise recommendation after 3 years. Adherence to physical activity recommendations in HF patients is generally found to be low, which may limit the effect on clinical outcomes, such as HF readmission and mortality [4, 7, 8]. There are many factors that influence adherence in general, and adherence to physical activity in particular. Self-efficacy and motivation are important aspects in being and staying physically active, and to overcome barriers [9, 10].

To improve adherence to exercise in HF patients, alternative approaches to motivate and increase self-efficacy to exercise are needed. A recent scoping review of health game research showed a constant growth over the past few years and a positive progress towards adapting new technology in specialised health contexts. Most health game studies address physical activity (28%), including exergames (games to improve physical exercise) [11]. A meta-analysis of energy expenditure in exergaming showed that playing exergames increases heart rate, oxygen uptake and energy expenditure from resting, and may facilitate the promotion of light to moderate physical activity [12]. Exergames might also be an option for HF patients to increase physical activity at home and encourage them to exercise, especially among people who may be reluctant to engage in more traditional forms of exercise, such as going to the gym or taking a walk outside. However, until now, most studies on exergaming among adults with systematic disabling conditions have only included male stroke survivors [13]. Except for one case study [14], no studies have been conducted on exergaming in HF patients. We therefore conducted a home-based physical activity intervention pilot study. The aims of the study were to assess the influence of the exergame platform Nintendo Wii on exercise capacity and daily physical activity in heart failure patients, to study factors related to exercise capacity and daily physical activity, and to assess patients’ adherence to exergaming.

*Research questions:*

1. Does an intervention including instructions on how to use Wii and a Wii console at home increase the exercise capacity in HF patients, and what factors are related to the exercise capacity and change in exercise capacity over 12 weeks?
2. Does an intervention including instructions on how to use Wii and a Wii console at home increase the daily physical activity in HF patients, and what factors are related to the daily physical activity and change in daily physical activity over 12 weeks?
3. What factors are related to the time playing Wii in HF patients and change in time spent playing over 12 weeks?

## Methods

### Study design

A 12-week pilot study with a pretest-posttest design was conducted in Sweden between May 2012 and August 2013. A 12-week intervention period was chosen as this was considered to be a sufficient timeframe to observe changes in exercise capacity following exergaming at home [15].

### Participants and recruitment

Patients were recruited from a HF clinic at a county hospital in Sweden. Inclusion criteria were being diagnosed with symptomatic HF in New York Heart Association (NYHA) class II-IV, and being in a stable condition. Stability was defined as the absence of hospital admissions and emergency department visits in the last month. Patients younger than 18 years of age were excluded, but there was no upper age limit. Other exclusion criteria were documented problems with mobility, balance or sight, severe cognitive dysfunction or other severe psychiatric illness, anticipated short survival, and difficulties understanding or reading the Swedish language. Eligible patients who were believed to be interested in the study were approached by HF nurses. The study complied with the Declaration of Helsinki and was approved by the Regional Ethics Committee (2010/412-31). All patients provided informed consent prior to taking part in the study.

### Intervention

In this study we tested the exergame Wii (Nintendo Company Ltd., Kyoto, Japan), a platform with a wireless controller (the Wii Remote: 148 × 36.2 × 30.8 mm), which connects to the Wii console (159 × 44 × 216 mm) through Bluetooth. The Wii remote enabled patients to interact with the Wii console through movements. The patients were given the game Wii Sports, which includes bowling, tennis, baseball, golf, and boxing games.

The patients learned how to use the Wii during a one-hour instructor-led introduction session at the hospital. The Wii console was installed in the patients’ home one week after the introduction, and an instructor demonstrated how to use it once more. After the installation, the patients were encouraged to play exergames on their own or with others for 12 weeks. Safety guidelines were discussed and provided in writing after the installation (e.g., use the wrist strap during exergaming, stand at least 1 metre from the television, make sure that furniture, objects, and people are out of the play area). Patients were advised to exergame for 20 minutes per day, and they could increase their time playing if they felt good. During the 12 weeks the instructor was available for questions and telephone guidance for two hours per day during workdays. In case of medical problems, the patient was instructed to call the HF nurse. After finishing the study after 12 weeks, the patients were offered to buy or return the Wii.

### Procedures and measurements

HF nurses collected baseline data before the introduction session, and there was another data collection at a follow-up visit in the HF clinic after 12 weeks. The patients completed questionnaires and performed a six-minute walking test at baseline and after 12 weeks. They received an activity monitor at the introduction session, and the week before the home installation served as baseline data. They also received 12 weeks of daily diaries when their Wii was installed.

### Main variables

*Exercise Capacity* was assessed by a six-minute walking test (6MWT) at baseline and 12 weeks after having gained access to Wii. The 6MWT is a simple, low-cost method for estimating exercise capacity; only a pre-measured level surface and a timing device are needed [16]. The mode of exercise (walking) is familiar to patients, although it may represent a maximal test for some. The test has appeared to be useful for assessing other interventions, such as cardiac resynchronisation, and has strong predictive power for both mortality and morbidity. A 30 metre difference in the 6-MWT (based on self-rated physical function) between baseline and 12 weeks was considered clinically relevant [17]. A recent study confirmed that the minimal difference of importance for 6-minute walk test distances among patients with chronic heart failure is around 36 metres [18].

*Daily physical activity* was measured using the activity monitor DirectLife Triaxial Accelerometer for Movement Registration (TracmorD) (Philips New Wellness Solutions, Lifestyle Incubator, the Netherlands), which measures daily physical activity by registering body acceleration in 3 directions: up and down, side to side and front to back. These registered body accelerations were translated into kilojoule (kJ), taking age, gender, height and weight into account [19]. Daily physical activity was measured daily for 12 weeks. Patients wore the device in their pocket or as a necklace and they only had to wear it during the day.

*Time playing Wii* was measured with a daily diary, where the patients were asked to report the number of minutes they played every day for 12 weeks.

### Related factors

*Heart failure symptoms* (fatigue, shortness of breath) when playing Wii were measured through a daily diary for 12 weeks, with a numeric rating scale ranging from 10 (worst experienced fatigue, shortness of breath), to 0 (no experienced fatigue or shortness of breath whatsoever).

*Perceived physical effort* in relation to playing Wii was measured in the diary with the Borg's Rating of Perceived Exertion (RPE). The RPE is a valid and reliable instrument based on a subjective feeling of exertion and fatigue during exercise, and it is used to assess and regulate exercise intensity. The patients were asked to give a numerical value on a scale from 6 (no exertion at all), to 20 (maximum effort) (16).

*Self-efficacy* was measured with the Exercise Self Efficacy questionnaire (SEE) at baseline and 12 weeks. It was used to assess the self-efficacy beliefs specifically related to how confident patients felt about keeping up their exercise when facing potential barriers, such as a busy work schedule, physical fatigue, boredom, minor injuries, other time constraints, and family and home responsibilities. The SEE consists of six situations that might affect exercise participation. For each situation, the patients used a scale ranging from 0 (not confident), to 10 (very confident) to describe how confident they were about being able to exercise for 20 minutes, 3 times per week. The instrument is valid and reliable [9].

*Motivation* was measured at baseline and 12 weeks with the Exercise Motivation Index (EMI). The EMI is a valid and reliable instrument assessing participation motives in order to examine issues such as the influence of motives on exercise participation, how motives might influence the choice of activities, how affective responses to exercising may be influenced by reasons for exercising, and how involvement in physical activity might have a reciprocal influence on participation motives. The EMI consists of 15 statements; each statement is followed by a five-point verbal ranging from 0 (not important), to 4 (extremely important) (10).

*Anxiety and Depression* was assessed with the Hospital Anxiety and Depression Scale (HADS). The HADS is a valid and reliable instrument used to assess the prevalence of emotional distress among HF patients. The scale consists of 14 items in two sections, where 7 items measure anxiety (HADS-A), and the remaining 7 items depression (HADS-D). These are rated on a 4-point scale with different response options for each question, and with a theoretical range of 0 and 21 in each group [20].

Data on age, gender, socio-economic status, marital status, family composition, living situation, smoking, and alcohol consumption were collected through a questionnaire at baseline and 12 weeks. Data on clinical variables were collected from the patients’ medical charts.

### Data analysis

Statistical analyses were performed with the Statistical Package for the Social Science version 20 (SPSS, Chicago, Illinois). In the descriptive analyses, means and standard deviations were calculated for continuous data, and absolute numbers and percentages were computed for nominal variables. Pearson correlation was used to assess the association between descriptive variables, perceived exertion, HF symptoms and time exergaming. Possible relations between the main variables (exercise capacity, daily physical activity, and time exergaming), and background variables, (perceived exertion, HF symptoms, self-efficacy, motivation, and anxiety and depression) were analysed by Kruskal-Wallis analysis, Student´s t-test and one-way ANOVA for unpaired data where appropriate. For paired data the paired-sample t-test was used. Differences were considered statistically significant at p < 0.05.

If there were more than 3 missing values per week in the data extracted from the activity monitor, that week was excluded from the analyses. If there were 3 days or less of missing values in a week, these values were replaced with the mean kJ/day the patient expended in that specific week. In this study, missing values were not replaced in the analyses of the other instruments, unless stated in the how to replace missing values in the guidelines of the instruments. We used intention to treat analysis with regard to the primary outcome (6MWT) and reported data of all patients. If data were missing, either due to drop out or problems performing the test, these patients were treated as ‘no increase’.

## Results

### Sample characteristics

Thirty-three patients were invited by the HF nurses to participate in the study. One patient was excluded due to motor disabilities, and one patient dropped out during the intervention. The latter had to work abroad much of the time and did therefore not have the time to participate in the study (male, 62 years old).

The mean age among the 32 patients was 63 ± 14 years. Ten patients (31%) were female (Table 1). The majority of patients, 90% (n =27), were married or in a relationship, 90% (n =29) had children, and 82% had grandchildren (n =23). Approximately half of the patients (47%, n =15) had been diagnosed with HF within the last year. Most of them were in NYHA II (71%, n =22), meaning that they were comfortable at rest, but that ordinary physical activity resulted in mild to moderate symptoms, such as tiredness, palpitation, or dyspnoea. Thirty-one percent of the patients suffered from more than one disease, 22% (n =7) had a normal body mass index (BMI), whereas 42% (n = 11) of the patients were overweight, and 36% (n =11) were considered obese. One patient called the nurses for medical problems (male, 74 years old). Due to exergaming, this patient had myalgia in his arms. This patient also gained 5 kilograms in weight in one week. This was further monitored by the nurses, but it was not necessary to adapt any medication [14]. Two patients contacted the instructor; one had difficulties changing the batteries of the Wii remote, and one had difficulties starting the console. The instructor informed the patient how to change the batteries over the telephone and visited the patient who had problems starting the console. After visiting this patient, there were no other problems with the device.

**Table 1:** **Demographic and clinical variables**

	Total group
	N = 32
**Age (years)**	63 (±14, 29–88)*
**Female gender**	10 (31%)
**Education**	
- Higher than high school	18 (57%)
**Marital status**	
- Married/in a relationship	27 (90%)
**Children**	29 (97%)
**Grandchildren**	23 (82%)
**New York Heart Association class (NYHA)**	
- NYHAII	22 (71%)
- NYHA III	9 (29%)
**Time after diagnosis**	
- Less than 1 year	15 (47%)
**Currently smoking**	1 (4%)
**Alcohol consumption**	
- One glass or less a week	15 (52%)
**Comorbidity**	10 (31%)
Body Mass Index (BMI)**	29 (±4)
**Overweight/obesity (BMI > 25)**	25 (78%)
**Anxiety baseline**	5 (±3)
**Depression baseline**	4 (±3)
**Self-efficacy baseline**	6 (±2)
**Motivation baseline**	2 (±1)
**6-minute walking test baseline (meters)**	500 (±93)

*n (±SD, Range).

**BMI, Body Mass Index.

Means and standard deviations were calculated for continuous data, and absolute numbers and percentages were computed for nominal variables.

### Exercise capacity

At baseline, the patients walked a mean distance of 501 ± 95 meters in the 6MWT. At 12 weeks this distance had increased to 521 ± 101 meters. A clinically significant difference of 30 metres difference was found in 53% of the 32 patients (n =17) between baseline and 12 weeks. In addition, one patient increased the distance walked, even though it was less than the significant clinical difference of 30 metres. In total, 9 patients (28%) decreased the distance walked (-69 m ±28 m). Furthermore, 5 patients could not do the 6MWT at 12 weeks due to medical problems; one developed lung cancer, one was too tired to walk, one experienced muscle damage in their hip and two had leg problems.

Comorbidity was significantly related to the number of metres walked in the 6MWT at baseline (427 ± 102, p-value = .014), compared to patients without comorbidity (baseline 524 ± 82). No others factors were found to be significantly related to exercise capacity at baseline.

Patients who increased their walking distance in the 6MWT were in a significantly lower NYHA class (78% NYHA II), and had been diagnosed for a significantly shorter period of time (56% had been diagnosed within the last year), compared to the patients who decreased their walking distance in the 6MWT (44% NYHA II, p-value = .021; 22% diagnosed within the last year, p-value = .043). No other factors were found to be related to the change in exercise capacity over the 12-week intervention period (see Table 2).

**Table 2:** **Differences between patients who increased in exercise capacity (6MWT) after 12 weeks’ access to the Wii compared to patients who decreased in exercise capacity**

	↓6MWT*	↑6MWT**	Significance
	N = 9	N = 18	p-value
**Age** (Years)	67 (±17)	61 (±13)	.370
**Female gender**	3 (33%)	7 (39%)	.580
**Education**			.109
- Higher than high school	4 (44%)	13 (72%)	
**Marital status**			.308
- Married/in a relationship	9 (100%)	16 (89%)	
**Children**	9 (100%)	17 (94%)	.480
**Grandchildren**	7 (78%)	14 (78%)	.782
**New York Heart Association class (NYHA)**			.021
- NYHAII	4 (44%)	14 (78%)	
- NYHA III	5 (56%)	4 (22%)	
**Time after diagnosis**			.043
- Less than 1 year	2 (22%)	10 (56%)	
**Smoking**	0	1 (6%)	.651
**Alcohol consumption**			.448
- One glass or less a week	4 (44%)	10 (56%)	
**Comorbidity**	3 (33%)	4 (22%)	.542
Body Mass Index (BMI)***	27 (±5)	30 (±4)	.138
**Overweight/obesity (BMI > 25)**	5 (55%)	15 (93%)	.132
**Anxiety baseline**	7 (±4)	5 (±3)	.088
**Depression baseline**	4 (±2)	3 (±3)	.492
**Self-efficacy baseline**	6 (±2)	6 (±2)	.667
**Motivation baseline**	2 (±1)	2 (±1)	.199
**Mean time exergaming per day (minutes)**	28 (±15)	27 (±14)	.910

*↓ Decrease.

**↑ Increase.

**BMI, Body Mass Index.

The data was analysed by Kruskal-Wallis analysis, Student´s t-test where appropriate, means and standard deviations were calculated for continuous data, and absolute numbers and percentages were computed for nominal variables.

HF symptoms and perceived physical effort were not related to the time spent exergaming (HF symptom fatigue r = -.147; HF symptom shortness of breath r = -.278; perceived physical effort r = -.215).

### Daily physical activity

**Figure 1:**
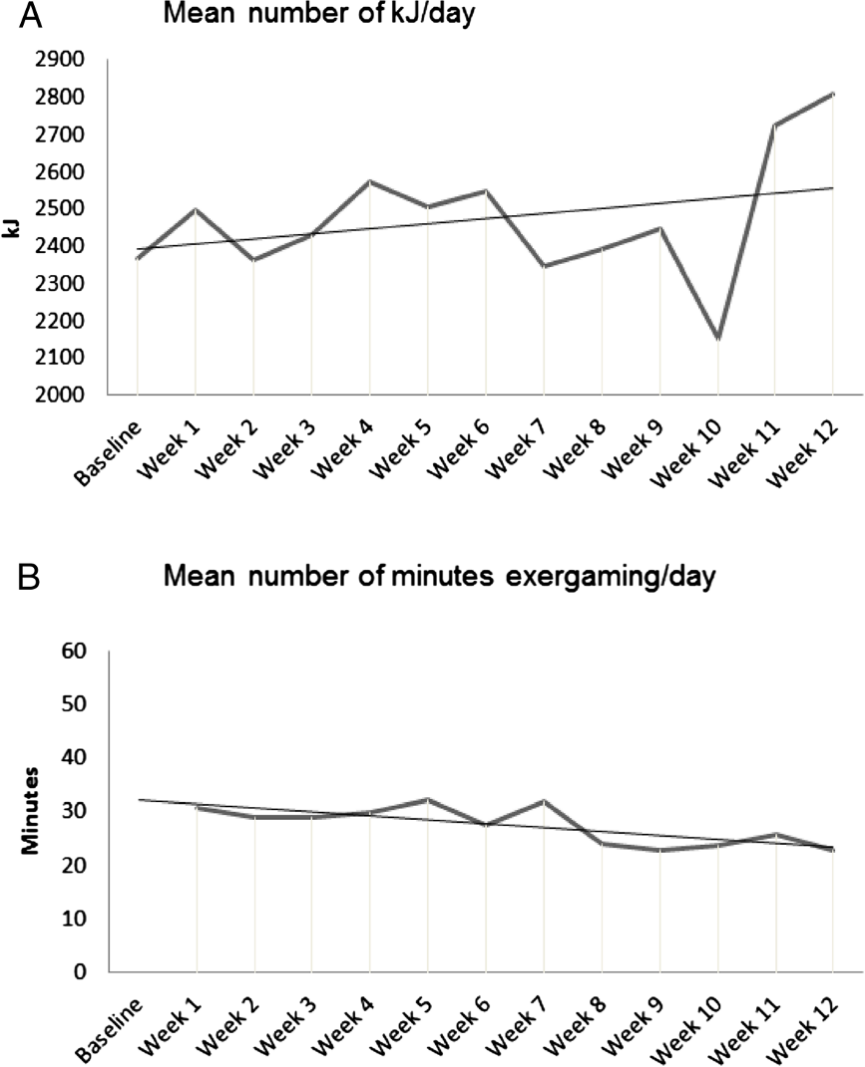
**Mean amount of Kilo Joules expended a day for each of the 12 weeks in the study (A) and the mean amount of minutes exergaming a day for each of the 12 weeks in the study (B).**

At baseline, the patients expended 2, 368 ± 847 kJ/day, and at 12 weeks they expended 2, 807 ± 1, 807 kJ/day, with no significant difference between those weeks (p-value = .291) (see Figure 1). Figure 1 shows a trend towards a gradual increase in energy expenditure, with the amount of energy expended fluctuating over the 12-week study period. No factors were found to be related with the number of kJ the patients expended a day, or the change in kJ expended over the 12-week intervention.

### Time spent playing Wii

The mean time spent exergaming was 28 ± 13 minutes per day (median of 27 minutes) (see Figure 1). Two patients stopped exergaming during the study, without giving a reason (both female, 63 and 57 years old). On average, male patients played Wii more minutes per day (32 ± 12 minutes) than female patients (19 ± 11, p-value = .033), while there was no significant difference in age between the men (68 ± 14) and the women (58 ± 14, p-value = .086). When the first 6 weeks of access to Wii were compared to the last 6 weeks, we found that both male and female patients had decreased the time spent exergaming. No gender differences were found regarding the decrease in number of minutes spent exergaming (-5 ± 9), however. Patients who played longer than the median time more often had grandchildren than patients who exergamed less than the median time (p-value = .024). No other factors were related to time spent playing Wii (see Table 3). The data showed that patients who played longer than the median time were more often married or in a relationship (p-value = .082), and had a lower educational background (p-value = .066) than patients who exergamed less than the median time. Figure 1 shows that the number of minutes played per week fluctuated over the 12-week study period, and that patients gradually decreased the number of minutes exergaming, although they still remained above the advised 20 minutes per day.

**Table 3:** **Differences between patients who increased in the number of minutes spent exergaming per day after the 12-week intervention compared to patients who decreased in the number of minutes spent exergaming per day**

	↓minutes exergaming than median N = 15*	↑minutes exergaming than median N = 15**	Significance
			p-value
**Age** (Years)	63 (±15)	65 (±14)	.370
**Female gender**	7 (47%)	3 (20%)	.160
**Education**			.066
- Higher than high school	12 (80%)	6 (40%)	
**Marital status**			.082
- Married/in a relationship	12 (80%)	14 (93%)	
**Children**	14 (93%)	14 (93%)	.334
**Grandchildren**	10 (67%)	13 (87%)	.024
**New York Heart Association class (NYHA)**			.392
- NYHAII	11 (73%)	9 (60%)	
- NYHA III	4 (27%)	5 (33%)	
**Time after diagnosis**			.472
- Less than 1 year	8 (53%)	6 (40%)	
**Smoking**	0	1 (7%)	.283
**Alcohol consumption**			.516
- One glass or less a week	9 (60%)	6 (40%)	
**Comorbidity**	3 (20%)	5 (33%)	.417
Body Mass Index (BMI)***	30 (±5)	28 (±4)	.284
**Overweight/obesity (BMI > 25)**	12 (80%)	11(74%)	.671
**Anxiety baseline**	5 (±3)	6 (±4)	.437
**Depression baseline**	4 (±3)	3 (±2)	.440
**Self-efficacy baseline**	6 (±2)	6 (±2)	.610
**Motivation baseline**	2 (±1)	2 (±1)	.944
**6 minute walking test baseline (meters**	503 (±98)	493 (±95)	.770
**6 minute walking test 12 weeks (meters)**	516 (±115)	525 (±87)	.804
**Delta 6 minute walking test (meters)**	9(±70)	33(±78)	.402

*↓ Decrease.

**↑ Increase.

**BMI, Body Mass Index.

De was data analysed by Kruskal-Wallis analysis, Student´s t-test where appropriate, means and standard deviations were calculated for continuous data, and absolute numbers and percentages were computed for nominal variables.

## Discussion

This is the first study that reports on experiences of elderly HF patients using a Wii console. The results of the study confirm the conclusion from an earlier scoping review, that exergaming might be a new alternative for rehabilitation in HF patients [15]. Out of the 32 patients included in this study, more than half showed a clinically significant increase in the 6MWT after 12 weeks’ access to Wii. This demonstrates that access to a Wii console at home could be a new method for increasing exercise capacity in HF patients. Patients did not increase their daily physical activity, which suggests that the increase of exercise capacity did not generate more activity in daily life.

The study also showed that this approach is feasible (e.g., all patients could learn to play, no major problems occurred, most patients continued to play), and might be effective for a large group of HF patients, independent of age, gender and comorbidity. The time period following the HF diagnosis, and NYHA class were related to the change in exercise capacity over the 12 weeks. This indicates that the longer a patient suffers from HF and the sicker the patient is, the more difficult it becomes to increase exercise capacity using this type of exercise intervention. An interesting finding was that the time exergaming was related to having grandchildren, which suggests that social facilitation could increase the time spent exergaming. A social environment that is not interested in physical activity is known to be a barrier for physical activity in HF patients. Exergaming could facilitate a greater connectedness with family, especially grandchildren, and pull down some of the barriers for becoming physically active. This result has also been reported in previous studies about exergaming in older adults [21, 22].

An additional finding was that HF symptoms and perceived physical effort were not related to the time spent exergaming, indicating that the patients did not perceive the activity as very strenuous or that it increased their HF symptoms. It was also reassuring that exergaming seemed safe and did not cause injury, and did not increase HF symptoms or perceived physical effort. In total, 17 of the 32 included patients increased their exercise capacity and 9 patients decreased. Using the recommended 30 metre increase (based on self-rated physical function) as a gauge for clinical significance is rather strict, and its usefulnesscan be discussed [16, 17]. Heart failure patients are known to deteriorate over time, and an intervention might even be found to be successful if a patient does not deteriorate over time.

Notably, but not surprisingly, there was a difference between men and women with regard to the time spent exergaming. This result is supported by the literature, where men generally favour exergaming more than women [12]. This might imply that in the future, men and women’s preferences should be examined. However, this should not be overstated, as both men and women played longer than the time advised by the nurses. In addition, women decreased as much as men in the time spent exergaming during the 12 weeks they had access to Wii. As the number of minutes spent exergaming fluctuates in the study, and as there was a small decrease at the end, it could be advised that future interventions have more follow-up points in order to keep the time spent exergaming the same [21].

This study shows a trend towards increased energy expenditure, where the largest change in energy expenditure occurred in week 11 and 12. A reason for this change could be the telephone contact in week 11, where patients were invited to visit the hospital for the follow-up measurement, which might triggered them to be more active.

Previous studies have shown that there is a difference in the amount of energy expended for different exergames. Bowling expended the least amount of energy and tennis and boxing the highest amount [12]. This provided the patient with the option to either increase/decrease their energy expenditure by changing exergames.

It should be noted that we think that familiarisation with Wii in the hospital, repeated instructions to the exergames during the installation of Wii at home, and the opportunities for further guidance in the first 12 weeks were crucial for the amount of time HF patients exergamed in this study. This is also supported by other research [22–24].

There are some methodological considerations in this study. First, the patients were selected by the nurses. Therefore, there is a possibility that the patients included were a motivated group. Second, this was a pilot study and there was no control group. The mechanism underlying the patients’ increase in the 6-minute walking test needs to be carefully interpreted. In this small group we found no difference in time exergaming between the group who increased in exercise capacity and the group who decreased in exercise capacity. However, other mechanisms (increased confidence, increased performance of other activities) might be hypothesised to result in better outcomes. Patients might have played different games with different energy expenditure. If these data were missing in the 6-minute walking test, we treated the patients as ‘no increase’. We realise that that these patients could have decreased in exercise capacity and, therefore, this is a limitation in this study. Another methodological consideration is the placement of the activity monitor on the patients. In the validation study of the activity monitor, the device was placed on the lower back, using an electric belt. In this study, the patients wore the device as a necklace or in their pocket, as advised by the manufacturer. This means that the device was not tightly fixed to the body and there is a possibility that some of the movements registered did not reflect body movement.

## Conclusion

It can be concluded that instruction on how to use Wii and access to a Wii console at home for 12 weeks increased the exercise capacity in more than half of the patients in this study. Lower NYHA class and shorter time since diagnosis was related to lower exercise capacity. We also found a non-significant trend towards an increase in daily physical activity after gaining access to the Wii console. The patients exergamed more minutes per day than the advised 20 minutes (mean minutes/day 28 ± 13). Having grandchildren and being male were related to more time exergaming.

There is a need for further research, with higher methodological quality to examine the effect of a home-based exergaming intervention aiming to increase exercise capacity. Therefore, a RCT study is currently being conducted (clinicaltrial.gov NCT01785121).
